# Inhibition of urokinase-type plasminogen activator expression by dihydroartemisinin in breast cancer cells

**DOI:** 10.3892/ol.2014.1918

**Published:** 2014-02-27

**Authors:** SHUQUN ZHANG, YINAN MA, JIANTAO JIANG, ZHIJUN DAI, XIAOYAN GAO, XIAORAN YIN, WENTAO XI, WEILI MIN

**Affiliations:** Department of Oncology, The Second Affiliated Hospital of Xi’an Jiaotong University, Xi’an, Shaanxi 710004, P.R. China

**Keywords:** breast cancer, urokinase-type plasminogen activator, dihydroartemisinin

## Abstract

The aim of the present study was to investigate the inhibitory effects of dihydroartemisinin (DHA) on the primary tumor growth and metastasis of the human breast cancer cell line, MDA-MB-231, *in vitro*. The expression levels of urokinase-type plasminogen activator (uPA) were detected by immunocytochemistry in two cell lines (MCF-7 and MDA-MB-231). The MDA-MB-231 cell activity was inhibited by various concentration gradients of DHA. The inhibitory rate, cell growth curve and apoptotic morphological observations were obtained using the MTT assay at 0, 24, 48 and 72 h. Cell scratch migration was performed at various time-points to test the cell proliferation and migration capacity. Reverse transcription-polymerase chain reaction was used to analyze the effect of DHA on uPA mRNA expression in breast cancer cells. The human breast cancer cell line, MDA-MB-231, possesses higher metastatic potential and relatively higher expression of uPA when compared with the MCF-7 cell line. DHA was found to inhibit the proliferation and migration capacity of the cell line, MDA-MB-231, *in vitro*. The growth inhibition occurred in a time- and dose-dependent manner, with IC_50_ values of 117.76±0.04, 60.26±0.12 and 52.96±0.07 μmol/l following 24, 48 and 72 h, respectively. The inhibition of uPA was observed to decrease breast cancer cell growth and migration. Thus, results of the present study indicate that DHA may be used for further studies with regard to breast cancer therapy.

## Introduction

Invasion and metastasis are the main biological characteristics of malignant tumors, which are considered lethal factors in the majority of cancer patients. The degradation of the basement membrane and the extracellular matrix (ECM) is a key step in this process. Urokinase-type plasminogen activator (uPA) is an essential protein that promotes invasion and metastasis. uPA is important for the hydrolysis of the basement membrane and ECM. A large number of previous studies have demonstrated higher expression of uPA in malignant tumor tissue and blood circulation components involved in tumor metastasis compared with normal tissues (1,2). uPA has been regarded as a marker of poor prognosis since the late 1980s. Multifactorial Cox model analysis previously revealed that a high level of uPA expression negatively correlates with disease-free and overall survival, but positively correlates with the risk of recurrence in breast cancer patients (2). High levels of uPA and/or plasminogen activator inhibitor-1 antigens in cytosolic extracts of human primary breast cancer tissue have been associated with rapid disease progression and lower overall survival (3). Mani *et al* (4) demonstrated that the small-molecule inhibition of the urokinase plasminogen activator receptor-uPA complex blocks breast invasion by MDA-MB-231 cells and inhibits matrix metalloproteinase-mediated ECM breakdown. The uPA system is regarded as an independent factor for predicting the prognosis of breast cancer and its significance is similar with that of the armpit lymph node (5).

Artemisinin (ART) is a natural sesquiterpene lactone from *Artemisia annua* with an endoperoxide group. ART and its derivatives are widely used as antimalarial drugs without obvious side effects. Previous reports of its antimalarial properties date back to 300 B.C., when ART was used as a traditional Chinese medicine for fever and chills. The anti-cancer properties of ART were first assayed *in vitro* in the late 1980s. Efferth *et al* (6) analyzed the anticancer activity of ART against 55 cell lines. ART acquires a highly active endoperoxide bridge once it encounters ferrous iron. Cancer cells express higher amounts of the transferrin receptor and consequently, have higher amounts of intracellular iron (7). Thus, these cells are prone to the intracellular production of reactive oxygen. The anticancer properties of ART have been extensively investigated and characterized in various experimental settings, including oxidative damage, apoptotic induction, cell cycle arrest, angiogenesis inhibition, aborted lymphatic metastasis and enhanced radiosensitivity (8–13). Dihydroartemisinin (DHA) is the main active metabolite of ART and its antimalarial and antitumor activities are stronger than those of the other ART derivatives. The high activity of DHA has been attributed to its higher water solubility, fine absorbency and excellent stability in clinical applications. Its efficient selective anticancer effects and lower toxicity have made DHA a novel research hotspot.

Cancer is a multifactorial ailment, thus, cancer therapy must target the various aspects of the disease. Considering the aforementioned factors, the classical concept of uPA and its associated fibrinolytic system have highlighted novel insights into the mechanisms of cancer progression. As described previously, DHA has strong anticancer properties. However, its ability to decrease the uPA levels in human breast cancer cell lines or weaken the metastatic ability of these cells remain unknown. In the present study, the potential mechanisms for the observed effects are presented. In addition, the aim of the present study was to investigate the antimetastatic effect induced by uPA and to highlight a molecular basis for the clinical use of DHA in the treatment of breast cancer.

## Materials and methods

### Cell culture

The highly metastatic MDA-MB-231 (purchased from Shanghai Cell Bank of Chinese Academy of Sciences, Shanghai, China) and the more common metastatic MCF-7 (courtesy of the Tumor Pathology Laboratory of the Xi’an Jiaotong University, Xi’an, China) breast cancer cell lines were used as the cancer cell models. These lines were cultured in Dulbecco’s Modified Eagle Medium (DMEM) supplemented with 10% fetal bovine serum and 1% antibiotic mixture (100 U/ml penicillin and 100 μg/ml streptomycin) in a CO_2_ incubator at 37°C with 5% CO_2_. This study was approved by the ethics committee of The Second Affiliated Hospital of Xi’an Jiaotong University (Xi’an, China).

### MTT assay

The MDA-MB-231 and MCF-7 cells were treated with various concentrations of DHA. Cell viability was measured using the MTT assay, which is based on the conversion of MTT to form crystals by mitochondrial dehydrogenase. Cells were plated at a density of 1×10^4^ cells/well in 96-well plates for 12 h prior to treatment with DHA or dimethyl sulfoxide (control) for 24, 48 and 72 h. In total, 20 μl MTT (5 mg/ml in phosphate buffered saline) was added to each well 4 h prior to the desired endpoint to dissolve the formazan crystals. The absorbance, which represented the optical density (OD), was measured at 570 nm in a 96-well plate reader (model no. 550; Bio-Rad, Hercules, CA, USA).

### Reverse transcription-polymerase chain reaction (RT-PCR)

uPA and GAPDH gene transcription levels were detected by RT-PCR using the following primer pairs: i) GAPDH forward, 5′-ACCCAGAAGACTGTGGATGG-3′ and reverse, 5′-TTCTAGACGGCAGGTCAGGT-3′ (590 bp); and ii) uPA forward, 5′-AGAATTCACCACCATCGAGA-3′ and reverse, 5′-ATCAGCTTCACAACAGTCAT-3′ (474 bp). The primers were synthesized by Beijing Aoke Biotechnology Co., Ltd. (Beijing, China).

Total RNA was isolated from cells using TRIzol reagent, according to the manufacturer’s instructions. The 20 μl PCR system contained 10X buffer (2 μl), cDNA (1 μl), 5 μmol/l forward primer (0.8 μl), 5 μmol/l reverse primer (0.8 μl), 10 mmol/l dNTPs (2 μl) and *Taq* DNA polymerase (1 units). The samples were first denatured at 94°C for 4 min prior to 30 PCR cycles of 94°C for 30 sec, 55°C for 56 sec and 72°C for 1 min, with an additional extension at 72°C for 10 min. The amplicons were visualized on 1.5% agarose gels. The negative controls were run as parallel experiments performed in the absence of cDNA. The GAPDH PCR product (200 bp) was used as an internal reference standard to compare the quantity of the cDNA template added to the PCR.

### Statistical analysis

Statistical analysis was performed using SPSS version 13.0 software (SPSS, Inc., Chicago, IL, USA). Data are expressed as mean ± standard deviation and P<0.05 was considered to indicate a statistically significant difference.

## Results

### Expression levels of uPA

**Figure 1:**
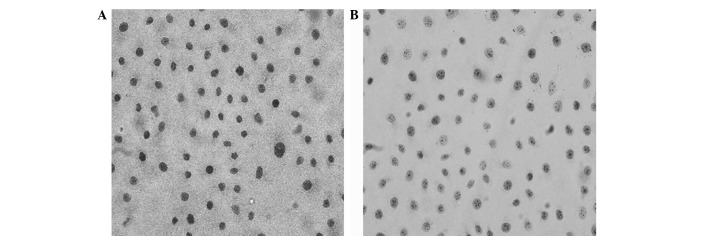
Expression of uPA in two subgroups of breast cancer cells; (A) MDA-MB-231 and (B) MCF-7. uPA, urokinase-type plasminogen activator.

Immunocytochemistry was used to detect the uPA expression in the human breast cancer cell lines, MDA-MB-231 and MCF-7. The various expression levels of uPA were observed using light microscopy following the immunohistochemical staining of samples from the two cell lines. Numerous deeply stained brown particles were observed on the membrane and in the cytoplasm of MDA-MB-231 cells (Fig. 1A). Compared with the MDA-MB-231 cells, the weakly stained MCF-7 cells exhibited sparse brown particles (Fig. 1B). In total, 10 fields-of-vision were randomly selected and 100 cells were counted in each field to calculate the positive cell index using the following formula: Positive cell index = (number of positive cells/1,000) × 100. The positive cell index was 63 and 24% in the MDA-MB-231 and MCF-7 cells, respectively. The Student’s t-test confirmed that the difference between the two values was statistically significant (P<0.01).

### Inhibition of cell growth by DHA

**Figure 2:**
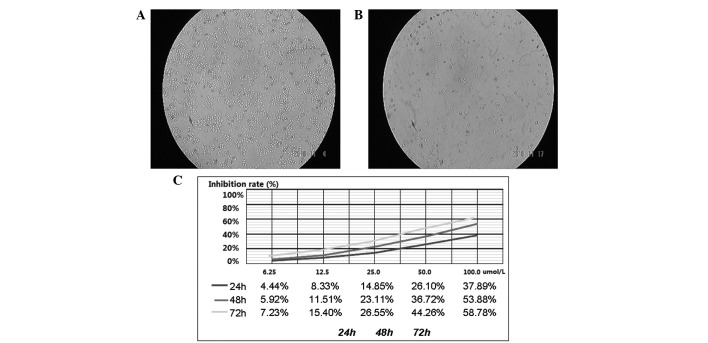
Inhibition of cell growth in MDA-MB-231 cells by DHA. Control and experimental group treated with 0, 6.25, 12.5, 25, 50 and 100 μmol/l DHA for 24, 48 and 72 h were observed and photographed by an inverted system microscope. (A) Control group, (B) experimental group. (C) Effects of DHA on breast cancer MDA-MB-231 cells. Cell viability was measured with an MTT assay. Y-axis, inhibition rate, was determined in assay and repeated a minimum of three times in duplicate. X-axis, various concertrations of DHA (0, 0 μmol/l; 1, 6.25 μmol/l; 2, 12.5 μmol/l; 3, 25 μmol/l ; 4, 50 μmol/l; 5, 100 μmol/l). DHA, dihydroartemisinin.

To assess the overall effect of DHA on the cellular growth, five doses of DHA were administered and compared. The MTT colorimetric assay demonstrated the viability of MDA-MB-231 cells in the experimental and control groups. The experimental group was treated with various concentrations of DHA for 24, 48 and 72 h and the control group was treated with DMEM in the same manner parallel to the intervention. The OD values were measured using MTT assay and analyzed statistically to draw the growth inhibition curve based on the half inhibition rate. The results showed that MDA-MB-231 human breast cancer cells were inhibited by DHA in a time- and dose-dependent manner. Specifically, as the DHA concentration was increased and its time of activity was extended, the inhibition rate was gradually increased, as detected by MTT in the MDA-MB-231 cells (Fig. 2). The inhibition rate was calculated based on half the IC_50_ value. The results showed that the inhibition rate of DHA on MDA-MB-231 was 117.76±0.04 μmol/l at IC_50 (24 h)_, 60.26±0.12 μmol/l at IC_50 (48 h)_ and 52.96±0.07 μmol/l at IC_50 (72 h)_ (Table I). These values were found to be statistically significant, as compared with the control group (P<0.05).

### RT-PCR

**Figure 3:**
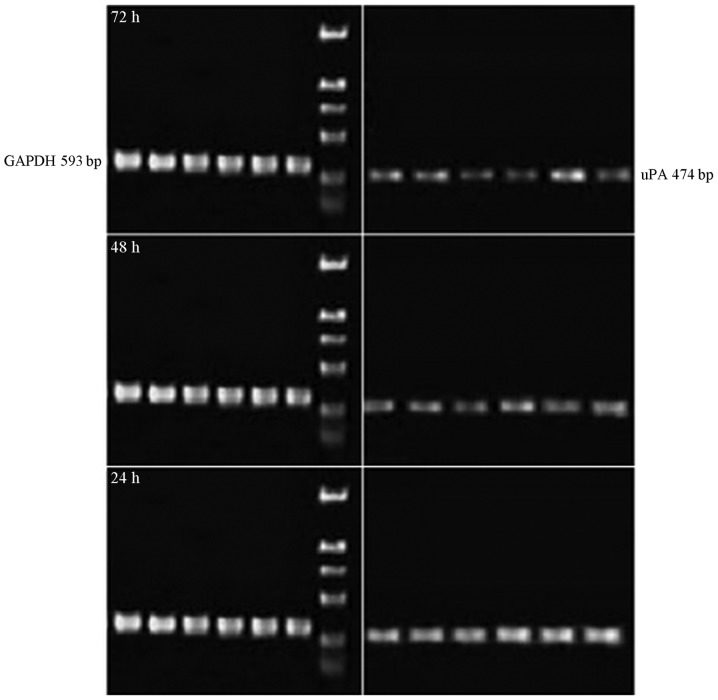
Electrophoresis results of reverse transcription-polymerase chain reaction amplification product. Left images present the bands corresponding to the internal control (GAPDH) and the right images show the target gene (uPA) at 72, 48 and 24 h. Six bands are shown for each gene. uPA, urokinase-type plasminogen activator.

**Figure 4:**
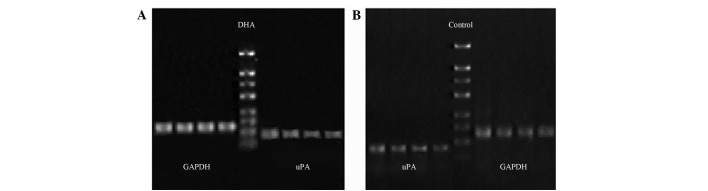
MDA-MB-231 cells were incubated with various concentrations of (A) DHA (experimental groups) and (B) equivalent amounts of Dulbecco’s Modified Eagle Medium (control group). The optical density ratio was calculated between the target (uPA) and reference (GAPDH) genes. uPA, urokinase-type plasminogen activator; DHA, dihydroartemisinin.

**Figure 5:**
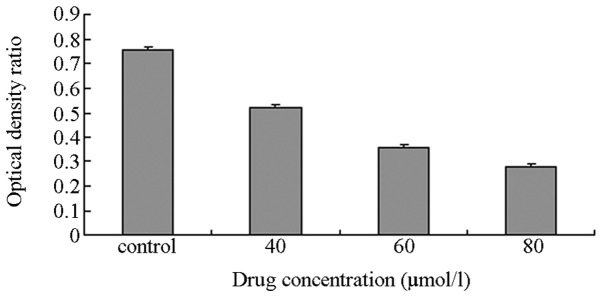
Graph presenting the optical density ratio between the target (urokinase-type plasminogen activator) and reference (GAPDH) genes.

The IC_50 (48 h)_ value was 60.26±0.12 μmol/l and was taken as the baseline concentration. Thus, 40, 60 and 80 μmol/l DHA were selected as the concentration gradients for the subsequent experiment. The MDA-MB-231 cells were incubated with various concentrations of DHA as the experimental groups, whereas an equivalent amount of DMEM was used for the control group. The total intracellular RNA was extracted for RT-PCR. The OD ratio was calculated between the target and reference genes. The results showed that the OD ratio of the controls was 0.76 (Figs. 3–5). The OD ratios of the experimental groups with DHA concentrations of 40, 60 and 80 μmol/l, were 0.52, 0.36 and 0.28, respectively. The Student’s t-test of the experimental groups against the control exhibited small P-values (P≈0.028), thereby, indicating that the differences between groups were statistically significant.

### Cell migration ability

**Figure 6:**
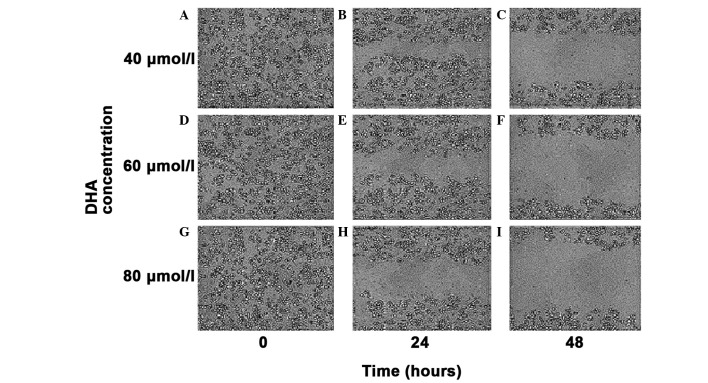
Cell scratch migration assay of the migratory ability of MDA-MB-231 cells. (A,B,C) Migration distance of the MDA MB 231 cells with a concentration of DHA of 40 μmol/l for 0, 24 and 48 h *in vitro*. (D,E,F) Migration distance of the MDA MB 231 cells with a concentration of DHA of 60 μmol/l for 0, 24 and 48 h *in vitro. (G,H,I)* Migration distance of the MDA MB 231 cells with a concentration of DHA of 80 μmol/l for 0, 24 and 48 h *in vitro.* Magnification, ×100.

The cell scratch migration assay evaluated the migrating ability of MDA-MB-231 cells following treatment with a 40, 60 and 80 μmol/l concentration gradient of DHA *in vitro*. At the selected times of 0, 24 and 48 h, the cell migration distance was observed under an inverted microscope (magnification, ×100) and images of the cells were captured (Fig. 6). As the drug concentration gradually increased, the migration distance of the MDA-MB-231 cells was progressively shortened, thereby, indicating the gradual decrease in the migrating ability of the MDA-MB-231 cells.

## Discussion

Breast cancer is one of the most common types of malignancy in females worldwide. As a systemic disease, metastatic breast cancer is considered incurable, with a median survival time of 2–3 years. Thus, the development of novel drugs or treatments to prolong survival or improve patient quality of life is urgently required. Such developments are likely to make it possible to maximize the efficacy but minimize the toxicity of these treatments. The uPA-centered fibrinolytic degradation system is important during the invasion and metastasis of breast cancer cells. The elucidation of its specific mechanisms of action are likely to allow for the possible development of effective drugs to block this system. Singh and Lai (14) previously found that DHA exhibited no evident cytotoxic effects on the normal breast HTB-125 cells, but was extremely toxic to the human breast cancer cell line, HTB-27. However, the toxicity of DHA may be improved by adding transferrin. Lai and Singh (15) reported that weekly oral intake of ART, at a dose of 10 mg/kg, was sufficient to retard breast cancer development in DMBA-treated rats. Li *et al* (16) confirmed that artesunate delayed liver metastases in nude mice with transplanted human breast cancer cells.

DHA is an ART analog that is well known for its excellent antimalarial ability. DHA is a form of traditional Chinese medicine, with the full intellectual property rights owned by the Chinese government. The anticancer properties of DHA have been gradually explored since its identification. However, the mechanism of its antimetastatic activity in breast cancer remains unclear. The current study assessed the effects of DHA on breast cancer cells and on tumor growth and invasion. The results showed that DHA reduces uPA expression and weakens the metastatic ability of the breast cancer cell line, MDA-MB-231. To the best of our knowledge, this is the first report of an ART derivative that may decrease uPA levels in a human cancer cell line. However, the associated mechanisms require further study. The following mechanisms may explanation our results.

On a molecular level, the mitogen-activated protein kinase (MAPK) signaling pathways and the nuclear transcription factor, nuclear factor (NF)-κB, have important functions for regulating uPA gene transcription and protein expression (17). The p38-MAPK signaling pathway enhances the activity of the uPA promoter, which further strengthens the uPA protein expression, thereby*,* improving the invasion and metastatic ability of cells by activating the NF-κB expression. De Cremoux *et al* (18) further analyzed this mechanism at the gene level and found that the half-life of uPA mRNA was associated with cell activation by p38-MAPK signaling (phosphorylated p38) in the highly invasive MDA-MB-231 breast cancer cell lines. The authors identified that uPA expression and cell invasion ability were significantly reduced by transfecting SB203580 (a p38-MAPK signaling inhibitor) into the MDA-MB-231 cells. In addition to the urokinase system antagonist and inhibitor, novel drugs for inhibiting MAPK or NF-κB may be useful for the treatment of malignant tumors. Chen *et al* (19) previously found that DHA may decrease the NF-κB content of the pancreatic cancer cell lines, BxPC-3 and AsPC-1. In addition, Tan *et al* (20) detected MAPK-related protein expression using western blot analysis of the ovarian cancer cell lines, SKOV3 and OVCAR3, following treatment with DHA. The authors results showed that DHA reduced the phosphorylation levels of ERK1/2 and inhibited the p38-MAPK pathway. Hwang *et al* (17) reported that DHA blocked phosphorylation in the PKCα/Raf/MAPK pathway, downregulated NF-κB in fibrosarcoma HT1080 cells and further affected the cell migration ability.

Thse results indicate that DHA inhibits the phosphorylation of the MAPK pathway and downregulates NF-κB expression. However, the direct association between uPA and DHA has not been confirmed. The results of the present study show that DHA downregulates the expression of uPA mRNA in the MDA-MB-231 breast cancer cell line. Similarly, the OD ratio between the target and reference genes decreased gradually with the increasing concentration and extended reaction time. The cell scratch experiment further showed that DHA weakens the migration ability of cells *in vitro*. Therefore, the attenuated migration ability in cells pretreated with DHA indicates the involvement of the uPA system and highlights novel clues for further investigation of the use of DHA for breast cancer therapy.

## Figures and Tables

**Table I:** OD of MDA-MA-231 cells interfered with DHA.

	OD values at various time-points			
	
DHA, μmol/l	0 h	24 h	48 h	72 h
0.00	0.325±0.011	0.330±0.011	0.381±0.008	0.483±0.013
6.25	0.325±0.012	0.305±0.013	0.319±0.006	0.402±0.006
12.50	0.325±0.013	0.271±0.005	0.289±0.011	0.391±0.007
25.00	0.325±0.014	0.249±0.005	0.278±0.003	0.345±0.005
50.00	0.325±0.015	0.236±0.004	0.201±0.005	0.304±0.010
100.00	0.325±0.016	0.225±0.010	0.178±0.010	0.264±0.003

DHA, dihydroartemisinin; OD, optical density.
